# Behavioral Oscillations in Visual Attention Modulated by Task Difficulty

**DOI:** 10.3389/fpsyg.2017.01630

**Published:** 2017-09-26

**Authors:** Airui Chen, Aijun Wang, Tianqi Wang, Xiaoyu Tang, Ming Zhang

**Affiliations:** ^1^Research Center for Psychology and Behavioral Sciences, Soochow University, Suzhou, China; ^2^Department of Psychology, Soochow University, Suzhou, China; ^3^School of Psychology, Liaoning Normal University, Dalian, China; ^4^Liaoning Collaborative Innovation Center of Children and Adolescents Healthy Personality Assessment and Cultivation, Dalian, China

**Keywords:** visual attention, attention spotlight, psychophysics, behavioral oscillations, task difficulty, flexible mechanism

## Abstract

The spotlight of attention is full of discrete moments and operates periodically. Recently, it has been well-documented there were behavioral oscillations in visual attention, however, different periodicities were demonstrated. Task difficulty may be an important factor causing disagreement in attentional periodic patterns. The present study examined behavioral oscillations in visual attention during difficult and easy tasks. A modified high temporal resolution cue-target paradigm in which the cue-target stimulus onset asynchrony (SOAs) varied from 0.1 to 1.08 s in steps of 20 ms was used. The target was detected with the accuracy of 65% in the difficult condition and 75% in the easy condition. Oscillatory patterns were analyzed and observed in behavioral performance. A theta rhythm was visible in the difficult version. However, attention oscillation increased to a higher frequency in the easy version. Task difficulty was negatively related to power for all bands. Our findings suggest that the attention spotlight switched faster when the task was easy, while, it switched much more slowly when the task was difficult in order to obtain more information. A flexible mechanism for attention spotlight was demonstrated, and task demand modulated attention oscillations.

## Introduction

Visual system is confronted with large amounts of information in the environment. Despite this, we can live an effortless and well-ordered life. This ability is attributed to attention. Attention aids us to selectively focus on spatial locations, objects or features, prioritizing relevant information for enhanced processing while ignoring others, just like a spotlight (Posner, 1980), a Gaussian gradient (Downing and Pinker, 1985), or a zoom lens (Eriksen et al., 1990). However, the temporal structures of behavioral and neural representations of perception and attention have been ignored. Recent studies have strongly demonstrated that the spotlight of attention is full of discrete moments and operates periodically, regardless of whether attention focuses on a single location or multiple locations, which is called rhythmic sampling of visual attention (VanRullen et al., 2007; Busch and VanRullen, 2010; Landau and Fries, 2012; Fiebelkorn et al., 2013; Song et al., 2014; Landau et al., 2015; Dugué et al., 2015b, 2016; VanRullen, 2016b). The precise periodic structures were also revealed in a dynamic priming effect, which may be the cognitive basis of time perception (Elliott, 2014).

One of the most important issues of rhythmic attention is the frequency of sampling. However, this issue remains poorly understood, and the rhythm was different across studies. Behavioral oscillations in participants’ accuracy (ACC) suggested that attention periodicity was 8 Hz in a target detection task (Landau and Fries, 2012; Fiebelkorn et al., 2013). However, other researchers analyzed behavioral oscillations in participants’ reaction time (RT) and observed a much higher frequency (8–20 Hz) in a target discrimination task (Song et al., 2014). A previous electroencephalography (EEG) study found that the phase of 7 Hz EEG activity just before flash onset modulated detection of the threshold flash under attention condition (Busch and VanRullen, 2010). The wagon wheel illusion was based on attentional motion computation at approximately 13 Hz (VanRullen et al., 2005). These attentional cycles also exist during visual searches in monkeys and humans (Buschman and Miller, 2009; Dugué et al., 2015a,b). A macaque monkey study found that attention shifts correlated with 18–34 Hz neural oscillations in the frontal eye fields (Buschman and Miller, 2009). Adopting psychophysics and transcranial magnetic stimulation (TMS) methods in human subjects revealed that attention periodically processed multiple search locations at approximately 5–10 Hz (Dugué et al., 2015a,b).

Task difficulty may be an important factor causing disagreement in attentional periodicities. A difficult 50% threshold task was set up in some studies and 8 Hz rhythm was revealed in the time course of attention (Landau and Fries, 2012; Fiebelkorn et al., 2013). However, an easily completed task was used, thus the subjects’ average accuracy was 98%; an 8–20 Hz rhythm was found (Song et al., 2014). Two monkeys were well-trained in the easy search task, and their attention shifted at 18–34 Hz (Buschman and Miller, 2009). However, human subjects completed dual tasks, including a challenging search task and a probe detection task, and their attention worked at lower frequency bands (Dugué et al., 2015a,b). Task difficulty may modulate the amount of attention to the same stimuli, and it is measured and reflected in behavioral tasks and neuronal activity. Subjects’ detectability was enhanced during difficult tasks (Urbach and Spitzer, 1995), and the response strength of inferior temporal and V4 neurons increased (Spitzer and Richmond, 1991; Boudreau et al., 2006).

**FIGURE 1:**
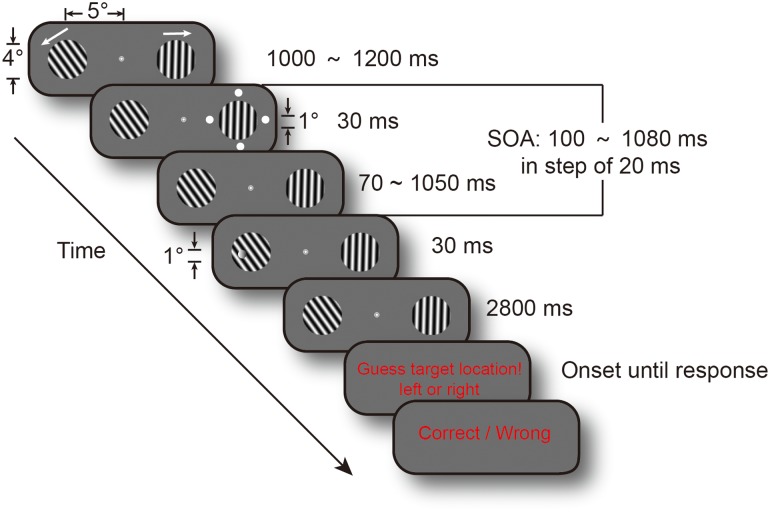
Experimental procedure. Each trial display contained two drifting gratings, and their drifting directions were randomized across trials. Cue events, consisting of four white dots, occurred at a random time between 1 and 1.2 s from fixation and gratings onset. Target could appear at one of the 50 temporal intervals, in steps of 20 ms, from 0.1 to 1.08 s after cue onset (SOA). Subjects were requested to determine target location, and their responses were recorded. The two arrows indicated gratings’ drifting directions.

The present study investigated whether task difficulty modulated attentional sampling behavior. We used a high temporal resolution cue-target paradigm to examine the temporal course of spatial attention. And, we used a threshold discrimination task rather than detection task because of decision biases in subjective criteria. **Figure 1** shows that subjects covertly attended to two gratings to discriminate the targets’ location in a two-alternative forced choice task (2AFC). Target discrimination performance in the difficult version was maintained at a 65% threshold by running a staircase procedure prior to the experiment. Target discrimination performance in the easy version was adjusted to a 75% threshold using the staircase method. Cue-to-target SOA ranged from 0.1 to 1.08 s, in steps of 20 ms, to achieve the temporal course of discrimination performance. The attention spotlight may switch much more slowly to obtain more information when the task is not easy and people have more difficulty in perceiving the stimulus if attention samples information from cluttered sensory environments in an intelligent and flexible manner. Thus, we predicted that manipulation of task difficulty would change the frequency of attention oscillations.

## Materials and Methods

Experiments were performed in accordance with the Declaration of Helsinki and approved by the ethical committee of Soochow University.

### Participants

Thirty-one subjects (26 females, age 18–26 years, all right-handed) were recruited to participate in the study. Sixteen subjects participated in the difficult task, and 15 subjects performed the easy task. All subjects had normal or corrected-to-normal vision. Participants gave informed consent prior to experiments in accordance with the Declaration of Helsinki and received monetary compensation afterward.

### Apparatus

Subjects sat 70 cm from a 22-inch ViewSonic P225f CRT monitor (1024 pixel × 768 pixel resolution at 100 Hz) in a dark room with their heads stabilized in a chin rest. The experiment was programmed using the Psychophysics Toolbox (Brainard, 1997; Pelli, 1997) in Matlab. Responses were recorded through a keyboard.

### Stimuli

The experimental scenario and parameters were adopted from Landau and Fries (2012). All stimuli were presented on a gray background (3.88 cd/m^2^). Subjects were requested to maintain fixation at a white bull eye (0.5° in diameter) and covertly monitor two gratings (4° in diameter) presented 5° eccentrically on either side of the fixation to identify the target’s location. The gratings, with spatial frequency 1.4 c/deg and contrast of 100%, drifted at 0.7 c/s. Drifting direction was randomized across trials (for reasons, see Landau and Fries, 2012, supplementary experimental procedures). The cue event, which consisted of four white disks (disk diameter: 1°; 1.5° away from the grating’s edge), was presented around either of the gratings for 30 ms. The target event was a contrast decrement of 30-ms duration, which randomly occurred on the left or right grating. The amplitude of contrast decrement was determined using a QUEST staircase procedure for each subject.

### Experimental Procedure

Fixation and gratings were presented at all times in each trial until red guess instruction onset. Cue events occurred randomly between 1 and 1.2 s from fixation and gratings onset. The target could appear at one of the 50 temporal intervals, in steps of 20 ms, from 0.1 to 1.08 s after cue onset (SOA) to achieve a continuous monitoring assay of subjects’ behavioral performance. The target randomly occurred on the cued side or the uncued side, with an equal likelihood of appearance. Subjects were informed of 50% cue validity prior to the experiment. Performances were evaluated in a 2AFC task. Subjects were instructed to judge the location of the target (left arrow for target on the left grating; right arrow for right grating) on the keyboard during a period of 2.8 s. Subjects who did not see the target guessed the location of the target and pressed either key. Visual feedback (correct or incorrect) was given after the subjects’ response. Each subject completed 1600 trials in total, in eight blocks on two or three separate days to avoid a fatigue effect.

### Staircase Procedure

The amplitude of target contrast decrement was determined using the QUEST procedure (Watson and Pelli, 1983), which determined the individual thresholds at which 65% (difficult task) or 75% (easy task) of the stimuli were detected. The staircase procedure was identical to the experimental procedure except that no cue stimuli were presented. Each block had 60 trials, and each experimental condition (65 or 75%) was measured three or four times.

### Data Analysis

Behavioral data were analyzed using Matlab and the CircStat toolbox (Berens, 2009). Performance accuracy was calculated as a function of SOA to represent temporal profiles for each condition (cued vs. uncued). Response time greater than 5 s was excluded from analysis. Spectral analysis was performed on temporal profiles from 0.1 to 1.08 s after cue onset to examine the spatiotemporal dynamics of the time course. We adopted the analysis method which Song and his colleagues used in their study to detrend the ACC time course for each condition (Song et al., 2014). Slow trends were obtained by calculating 200 ms moving of average-ACC temporal profile, and then we removed these trend signals from the 980 ms epoch. This epoch was Hanning tapered, padded with zeros and Fourier transformed. The phase relation between cued and uncued conditions was calculated. Phase angle between cued and uncued conditions for a single subject was analyzed as a function of frequency from 0 to 20 Hz. Between-subject coherence in phase angles was calculated and Rayleigh tests were executed for phase differences in the 4 ∼ 5 Hz band.

We used a non-parametric approach to assess the statistical significance of peaks in the spectra amplitude. Shuffling temporal profiles (ACC-SOA) separately within each subject 1000 times was performed to generate a randomization distribution. Surrogate signals were analyzed after each randomization in the same manner as the observed data. A permutation distribution for each frequency and significance thresholds (*p* < 0.05) were obtained.

## Results

Data in the difficult task were not normally distributed [*W*(15) = 0.821, *p* = 0.007]. Therefore, non-parametric statistical methods were used in the study to overcome the influence of outliers.

### Between-Subjects Analysis

Sixteen subjects performed a difficult task (65% version), and their average accuracy was 72.461% (*SD* = 10.012%). Fifteen subjects participated in an easy task (75% version), and their mean accuracy was 82.500% (*SD* = 6.996%). A Mann–Whitney *U*-test indicated that ACC was higher in the easy task, with a large effect size (*U* = 39.000; *N_1_* = 16; *N_2_* = 15; *p* = 0.001, *r* = 0.5751).

**FIGURE 2:**
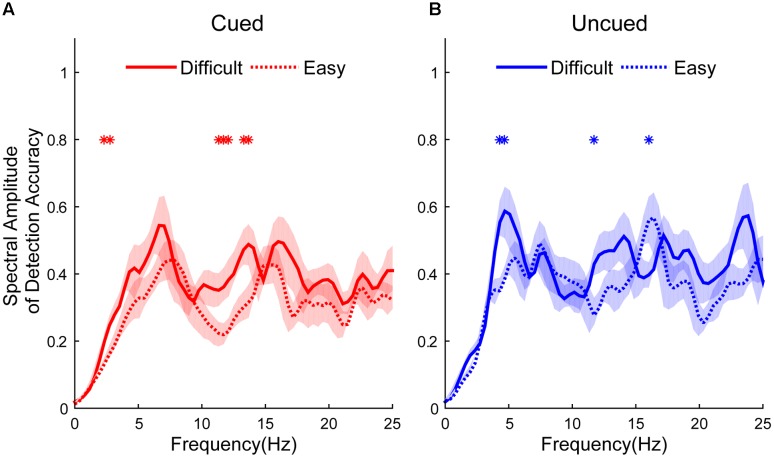
Spectral amplitudes of detection accuracy from difficult and easy tasks (difficult: solid line; easy: dashed line) between cued and uncued conditions (cued condition: red line, shown in **A**; uncued condition: blue line, shown in **B**). Red and blue asterisks indicate the significant peak frequencies between spectral amplitudes in the easy and difficult tasks.

**Figure 2** shows the different spectral amplitudes between easy and difficult conditions. The amplitudes of the easy task in the cued condition were significantly lower than in the difficult task at 2.3438, 2.7344, 11.3281, 11.7188, 12.1094, 13.2813, and 13.6719 Hz (independent *t*-test, *p* < 0.05, see **Figure 2A**). The amplitudes of the easy task in the uncued condition exhibited significantly lower amplitudes than the difficult task at 4.2969, 4.6875, and 11.7188 Hz but larger amplitudes at 16.0156 Hz (independent *t*-test, *p* < 0.05, see **Figure 2B**). No differences were found at other frequencies.

**FIGURE 3:**
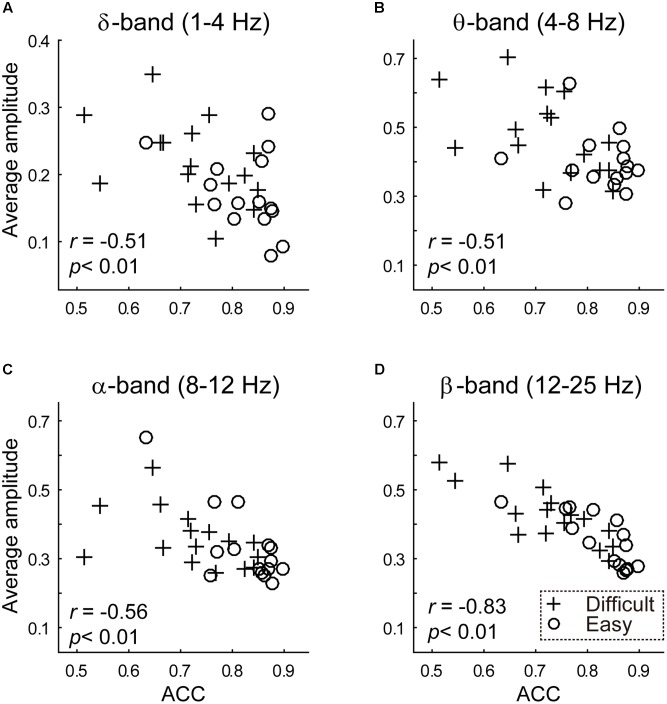
The results of between-subject analysis are plotted. The scatterplot between transformed **(A)** delta band (1 ∼ 4 Hz) **(B)** theta band (4 ∼ 8 Hz) **(C)** alpha band (8 ∼ 12 Hz) **(D)** beta band (12 ∼ 25 Hz) average amplitude (y-axis) and discrimination accuracy (x-axis). Average amplitudes were calculated using a Fast Fourier Transformation. Crossed and circles illustrated average amplitudes in the difficult and easy task, respectively.

Behavior performance was functionally related to the average amplitude of the delta-band (1 ∼ 4 Hz), theta-band (4 ∼ 8 Hz), alpha-band (8 ∼ 12 Hz) and beta-band (12 ∼ 25 Hz), as indicated by negative correlations (delta-band, *r* = -0.5095, *p* = 0.0034; theta-band, *r* = -0.5103, *p* = 0.0034; alpha-band, *r* = -0.5621, *p* < 0.001, beta-band, *r* = -0.8318, *p* < 0.001; see **Figure 3**).

### Within-Subject Analysis

#### Difficult Task

**FIGURE 4:**
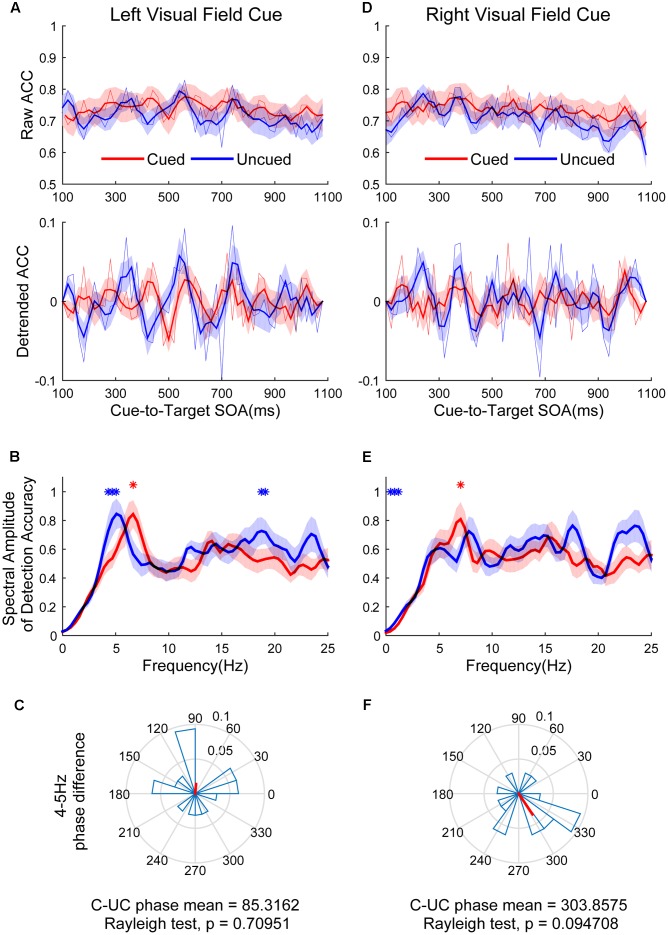
The results of ACC time course, spectral amplitudes and phase relationships between cued and uncued in difficult task. The left three figures represent data in the left visual field (LVF). **(A)** The raw ACC time course and detrended ACC time course as a function of cue-to-target SOAs under cued (red) and uncued (blue) conditions averaged across all subjects. **(B)** A frequency domain representation of the behavioural data shown in **(A)**. **(C)** Phase coherence between the cued and uncued conditions. Each subject’s phase difference is plotted on the circle, with the average difference plotted. **(D–F)** Represented when the cue event occurred in the right visual field (RVF), corresponding to **(A–C)**. Red and blue asterisks indicate the significant peak frequencies for cued and uncued relative locations.

**Figure 4** shows the raw ACC temporal courses as a function of cue-to-target SOAs under cued (red) and uncued (blue) conditions averaged across all subjects. **Figures 4A,D** show that a switching relationship between cued and uncued conditions was observed in the left visual field (LVF) and the right visual field (RVF). This result means that these two locations were being sampled alternatively, with better discrimination performance in one location and worse discrimination performance in the other location. Spectrotemporal analysis was performed separately in the left and right visual field to clarify temporal dynamics. Discrimination in the cued condition fluctuated at 6.6 Hz in the LVF (**Figure 4B**, red line; *p* < 0.05 for 6.6406 Hz) when the target occurred in the cued location. Discrimination in the uncued condition fluctuated at 4 and 19 Hz in the LVF (**Figure 4B**, blue line; *p* < 0.05 for 4.29688–5.07813 and 18.75–19.1406 Hz). A direct comparison between the peak frequencies for cued vs. uncued locations revealed no significant difference [*t*(15) = 1.357, *p* = 0.195]. The phase clustered around a mean of 85.3162 ± 74.7776° (**Figure 4C**). This phase was not significantly different from either 0 or 180°. Discrimination in the cued condition fluctuated at 7 Hz in the RVF (**Figure 4E**, red line; *p* < 0.05 for 7.0313 Hz) when the target occurred in the cued location. Discrimination in the uncued condition fluctuated at 0.5 and 1 Hz in the RVF (**Figure 4E**, blue line; *p* < 0.05 for 0.39063–0.78125 and 1.1719 Hz). A direct comparison between the peak frequencies for cued vs. uncued location revealed no significant difference [*t*(15) = 1.235, *p* = 0.36]. The phase of 4–5 Hz was clustered around a mean of 303.8575 ± 63.6608° (**Figure 4F**).

#### Easy Task

**FIGURE 5:**
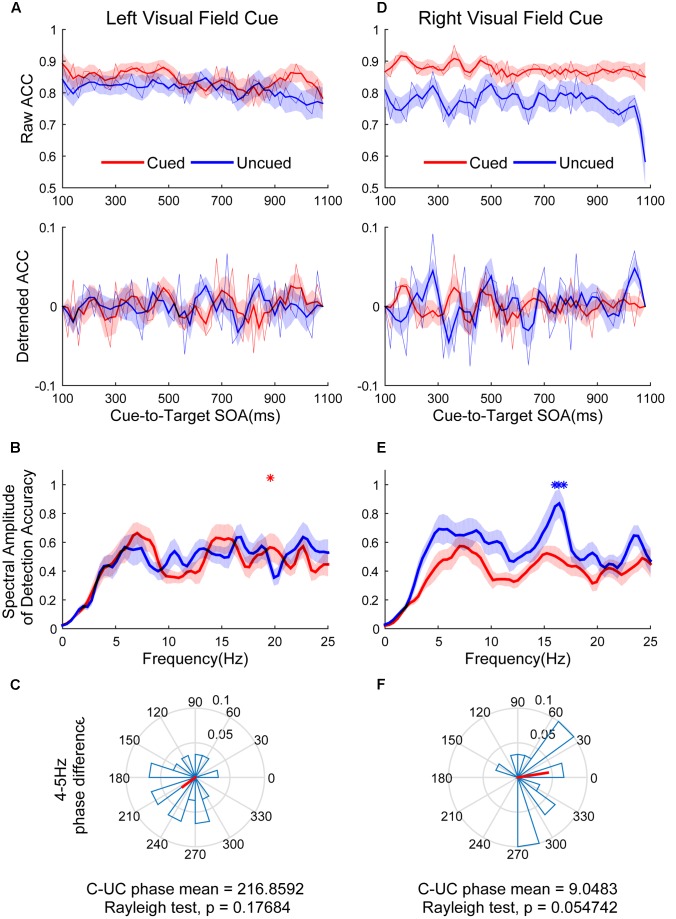
The results of ACC time course, spectral amplitude and phase relationships between cued and uncued in easy task. The left three figures represent data in the LVF. **(A)** The raw ACC time course and detrended ACC time course as a function of cue-to-target SOAs under cued condition (red) and uncued (blue) conditions averaged across all subjects. **(B)** A frequency domain representation of the behavioral data shown in **(A)**. **(C)** Phase coherence between the cued and uncued conditions. Each subject’s phase difference is plotted on the circle, with the average difference plotted. **(D–F)** Represent when the cue event occurred in the RVF, corresponding to **(A–C)**.

**Figure 5** shows the raw ACC temporal courses as a function of cue-to-target SOAs under cued (red) and uncued (blue) conditions averaged across all subjects. No significant switching relationship patterns between cued and uncued conditions were observed. Discrimination in the cued condition fluctuated at 19.5 Hz when the cue occurred in the LVF (**Figure 5B**, red line; *p* < 0.05 for 19.5313 Hz) and the target occurred in the cued location. No significant frequencies were found in the uncued condition (**Figure 5B**, blue line). A direct comparison between the peak frequencies for cued vs. uncued locations revealed no significant differences [*t*(14) = 0.052, *p* = 0.959]. These cycles exhibited a phase relationship that clustered around a mean of 216.8592 ± 65.7974° (**Figure 5C**). This result was not significantly different from either 0 or 180°. Discrimination in the uncued condition fluctuated at 16.4 Hz in the RVF (**Figure 5E**, blue line; *p* < 0.05 for 16.0156–16.7969 Hz). No significant fluctuation was observed in the cued condition. A direct comparison between the peak frequencies for cued vs. uncued locations revealed that the fluctuation of the uncued condition was stronger than the cued condition [*t*(14) = 2.571, *p* = 0.0226]. These cycles exhibited a phase relationship that clustered around a mean of 9.0483 ± 60.8117° (**Figure 5F**).

## Discussion

The present study used a high temporal resolution cue-target paradigm to examine the temporal dynamics of behavioral time courses in visual attention during difficult and easy tasks. We demonstrated that behavior performance alternated at cued and uncued locations at a theta rhythm in the difficult task version. Our findings present evidence of a covert attentional sampling process. We found a theta rhythm in the difficult task version, which is consistent with previous studies (Landau and Fries, 2012; Fiebelkorn et al., 2013) and suggests covert attention samples multiple locations periodically. The attention period increased to a high frequency in the easy task version. The results of between-subjects analysis demonstrated that task difficulty was related to power for the delta-band (1 ∼ 4 Hz), theta-band (4 ∼ 8 Hz), alpha-band (8 ∼ 12 Hz), and beta-band (12 ∼ 25 Hz). Specifically, subjects with better performance exhibited less power. The results of within-subjects and between-subjects analysis consistently demonstrated that task difficulty influenced the pattern of attention oscillations. The attention spotlight may switch faster when the task is easy, and it may switch much slower when the task is difficult in order to obtain more information. These results suggest that attention spotlight samples information from cluttered sensory environments in an intelligent and flexible manner.

The time-quantum model (TQM) assumes the existence of an absolute lower bound for intermittencies, the time-quantum, as an (approximately) universal constant that has a duration of approximately 4.5 ms. Any admissible intermittencies are integer multiples of the quantum (Geissler, 1987). Elliott found that the priming effect achieved strongest when the dynamic priming frequencies synchronized with 6.69 Hz (149, approximately 33 times of 4.5 ms) rhythm (Elliott, 2014). This author thought that the conscious realization of events was mediated sometimes by anticipatory cognition and sometimes by cognition, which occurred subsequent to the coded event. Our study demonstrated that the different oscillation pattern between difficult and easy tasks, thus indicated that attention oscillations were modulated by task difficulty. Dugué and colleagues found that search type modulated processing in a visual search. Alpha frequency predominated for feature search, and a theta frequency was observed for a conjunction search (Dugué et al., 2017). These results further suggest that attention and perception rhythm are not constant and may be modulated by anticipatory cognition, task difficulty, and other cognitive factors.

Neural oscillations in several brain regions and networks play important roles in perception and cognition (Buzsáki, 2006; Arnal and Giraud, 2012; Fries, 2015). Rhythmic frame by oscillations may force the sensory system to use the nested temporal structure within each oscillatory cycle. Concentrating neuronal resources at specific moments in time while sparing these resources at other time points may be economic (VanRullen, 2016a). We may assume that task difficulty mediates the allocation of neural recourses to influence attention oscillations in behavior. This speculation requires testing in further experiments.

Notably, we used a 2AFC task instead of a detection task to better avoid subjects’ criteria (conservative or risky strategy). Participants in the detection task reported “Did the target appear or not?” Subjects who adopted a riskier strategy would likely report that the target appeared, which created a high false alarm probability. Subjects who adopted a more conservative strategy tended to report that the target did not appear, which created low hit ratios (Drewes et al., 2015). Our improved methodology greatly avoided decision bias toward the oscillatory patterns. Previous studies used a detection task, and the present study used a 2AFC task. Both tasks found a 4-Hz oscillatory pattern in the 50% threshold version (Landau and Fries, 2012; Fiebelkorn et al., 2013). This result suggests our finding was the oscillating nature of attention rather than subjects’ decision fluctuations. Future studies should use more elaborate methods to investigate the mechanisms of attention. In the study, saccades or microsaccades were not monitored in our experiments. However, the results are likely not due to these exogenous attention effects. Saccades are executed as part of ongoing attentional rhythm instead of an inducer (Hogendoorn, 2016). The function of saccades or microsaccades generally manifest on the low frequency and cannot explain the high frequency of the easy version in the study.

Our findings suggest a flexible mechanism for attention spotlight that may be modulated by task demand. We speculate that task demand is akin to the brake pedal (slowdown) or accelerator pedal (speedup) of the attention sampling machine. Further studies should investigate when and where the discrete attentional sampling occurs in our visual hierarchy.

## Ethics Statement

The study was supported by Ethics Committee of Soochow University (ECSU). Firstly, the experimenter explained to participants and promised that the rights of individuals to privacy, confidentiality, and self-determination. Participants were informed that your participation in this research is entirely voluntary, and this experiment is totally harmless. Secondly, the experimenter explained what participants have to do in this research. Each participant gave written informed consent.

## Author Contributions

AC and MZ designed the research. AC and TW performed the research. AC analyzed the data. AC and AW wrote the manuscript text. MZ and XT reviewed the manuscript.

## Conflict of Interest Statement

The authors declare that the research was conducted in the absence of any commercial or financial relationships that could be construed as a potential conflict of interest.
